# Comparative genomic analysis of the tricarboxylic acid cycle members in four Solanaceae vegetable crops and expression pattern analysis in *Solanum tuberosum*

**DOI:** 10.1186/s12864-021-08109-9

**Published:** 2021-11-14

**Authors:** Yongming Liu, Jingtao Qu, Ziwen Shi, Peng Zhang, Maozhi Ren

**Affiliations:** 1 Institute of Urban Agriculture, Chinese Academy of Agricultural Sciences, 610213 Chengdu, China; 2 Zhengzhou Research Base, State Key Laboratory of Cotton Biology, School of Agricultural, Sciences of Zhengzhou University, 450000 Zhengzhou, China; 3 Hainan Yazhou Bay Seed Laboratory, 572025 Sanya, China; 4 Maize Research Institute, Sichuan Agricultural University, 611130 Chengdu, China

**Keywords:** TCA cycle, Solanaceae crops, Genome-wide identification, Citrate synthase, Subcellular analysis

## Abstract

**Background:**

The tricarboxylic acid (TCA) cycle is crucial for energy supply in animal, plant, and microbial cells. It is not only the main pathway of carbohydrate catabolism but also the final pathway of lipid and protein catabolism. Some TCA genes have been found to play important roles in the growth and development of tomato and potato, but no comprehensive study of TCA cycle genes in Solanaceae crops has been reported.

**Results:**

In this study, we analyzed TCA cycle genes in four important Solanaceae vegetable crops (potato (*Solanum tuberosum*), tomato (*Solanum lycopersicum*), eggplant (*Solanum melongena*), and pepper (*Capsicum annuum*)) based on comparative genomics. The four Solanaceae crops had a total of 180 TCA cycle genes: 43 in potato, 44 in tomato, 40 in eggplant, and 53 in pepper. Phylogenetic analysis, collinearity analysis, and tissue expression patterns revealed the conservation of and differences in TCA cycle genes between the four Solanaceae crops and found that there were unique subgroup members in Solanaceae crops that were independent of Arabidopsis genes. The expression analysis of potato TCA cycle genes showed that (1) they were widely expressed in various tissues, and some transcripts like Soltu.DM.01G003320.1(SCoAL) and Soltu.DM.04G021520.1 (SDH) mainly accumulate in vegetative organs, and some transcripts such as Soltu.DM.12G005620.3 (SDH) and Soltu.DM.02G007400.4 (MDH) are preferentially expressed in reproductive organs; (2) several transcripts can be significantly induced by hormones, such as Soltu.DM.08G023870.2 (IDH) and Soltu.DM.06G029290.1 (SDH) under ABA treatment, and Soltu.DM.07G021850.2 (CSY) and Soltu.DM.09G026740.1 (MDH) under BAP treatment, and Soltu.DM.02G000940.1 (IDH) and Soltu.DM.01G031350.4 (MDH) under GA treatment; (3) Soltu.DM.11G024650.1 (SDH) can be upregulated by the three disease resistance inducers including *Phytophthora infestans*, acibenzolar-S-methyl (BTH), and DL-β-amino-n-butyric acid (BABA); and (4) the levels of Soltu.DM.01G045790.1 (MDH), Soltu.DM.01G028520.3 (CSY), and Soltu.DM.12G028700.1 (CSY) can be activated by both NaCl and mannitol. The subcellular localization results of three potato citrate synthases showed that Soltu.DM.01G028520.3 was localized in mitochondria, while Soltu.DM.12G028700.1 and Soltu.DM.07G021850.1 were localized in the cytoplasm.

**Conclusions:**

This study provides a scientific foundation for the comprehensive understanding and functional studies of TCA cycle genes in Solanaceae crops and reveals their potential roles in potato growth, development, and stress response.

**Supplementary Information:**

The online version contains supplementary material available at 10.1186/s12864-021-08109-9.

## Background

The tricarboxylic acid (TCA) cycle, also known as the Krebs cycle, is the general term for a series of steps in the pyruvate oxidation process. Pyruvate undergoes oxidative decarboxylation to produce acetyl-CoA, which then enters the TCA cycle and is completely oxidized to CO_2_ and H_2_O. The TCA cycle is a common metabolic pathway through which carbohydrates, lipids, and proteins are completely oxidized in the body. It is not only the main way for the body to obtain energy but also the hub of the metabolic connection and interconversion of carbohydrate, lipids, proteins, and amino acids [1]. The TCA cycle consists of eight steps, which are catalyzed by eight different enzymes, namely, aconitase (ACO), isocitrate dehydrogenase (IDH), α-ketoglutarate dehydrogenase complex (αKGDHC), succinyl-CoA ligase (SCoAL), succinate dehydrogenase (SDH), fumarase (FUM), malate dehydrogenase (MDH), and citrate synthase (CSY). Among them, FUM, MDH and αKGDHC are likely targets for tricarboxylic acid cycle flux regulation [2]. Almost all the steps in the TCA cycle can be bypassed by reactions in other subcellular compartments, while the reactions catalyzed by citrate synthase and succinate dehydrogenase can only be carried out in mitochondria [3]. Further, most enzymatic reactions in the TCA cycle are reversible, except for the synthesis of succinyl-CoA and citric acid [1, 4]. In addition to participating in energy metabolism, the TCA cycle can function in cellular metabolic pathways such as photosynthesis, photorespiration, nitrate assimilation, and glycolysis [5].

In plants, various enzyme complexes of the TCA cycle are often composed of multiple proteins. Proteins in the same enzyme complex or different enzyme complexes can directly interact with each other to coordinate their catalytic functions [6]. At the same time, 125 interactions between subunits of the TCA cycle enzymes and other pathway enzymes or proteins have been revealed [7]. TCA cycle proteins have been shown to be directly regulated by diverse factors. Thioredoxin regulates the activity of mitochondrial CSY, SDH, and FUM by modulating their redox status [8, 9]. The transcription factor bZIP14 can directly bind the promoters of genes encoding succinate dehydrogenase flavoprotein and mitochondrial succinate dehydrogenase iron-sulfur subunit in response to nitrogen starvation in *Phaeodactylum tricornutum* [10]. Salicylic acid can bind the E2 subunit of mitochondrial α-ketoglutarate dehydrogenase to alter mitochondrial oxidative phosphorylation and electron transport chain components in tomato’s defense against *tobacco mosaic virus* (TMV) [11]. Phytochrome regulates the expression of the mitochondrial aconitase in maize leaves via affecting the promoter methylation level [12]. Moreover, posttranslational modifications such as phosphorylation and acetylation play regulatory roles in the TCA cycle [13, 14].

Solanaceae plants are one of the plant groups with the greatest economic value, providing humans with vegetables, medicines, and industrial raw materials [15, 16]. Due to their unique flavor and rich nutrition, Solanaceae vegetables such as potato (*Solanum tuberosum*), tomato (*Solanum lycopersicum*), eggplant (*Solanum melongena*), and pepper (*Capsicum annuum*) are very popular and can play important roles in alleviating food crises and supplying human nutrition.

In a previous study, we preliminarily identified the members of tomato TCA cycle [17]. The individual genes of each enzyme complex of the TCA cycle in tomato have been functionally analyzed. The *cis*-aconitase activity of aconitase 1 (*Aco-1*) mutants is reduced, the photosynthetic capacity of the leaves is increased, and the fruit yield is increased, but the flowering period is delayed, and the respiratory activity of the roots and dry matter accumulation are decreased [18]. Antisense inhibition of mitochondrial NAD-​dependent isocitrate dehydrogenase gene (*SlIDH1*) significantly reduces the TCA cycle flux and reduces the volume and weight of tomato fruits, while the levels of amino acids, TCA cycle intermediates, photosynthetic pigments, starch, and nicotinamide adenine dinucleotide/nicotinamide adenine dinucleotide phosphate (NAD(P)H) decrease slightly [19]. Silencing *SlICDH1* using virus-induced gene silencing (VIGS) in Micro-Tom tomatoes prolongs ripening and reduces susceptibility to the pathogen *Botrytis cinerea*, and the harvested fruits exhibit reduced respiration and ethylene production [20]. Antisense inhibition of the *E1* subunit of *SlOGDH* significantly reduces the respiration rate of the plant, which is accompanied by the phenomena of early flowering, accelerated fruit ripening, and premature leaf senescence [21]. Gene silencing and transient overexpression approaches have revealed that the *E2* subunit of *SlOGDH* plays a negative role in tomato defense against the virulent bacterial pathogen *Pseudomonas syringae* [22]. Tomato contains two genes encoding the SCoAL α subunit and one gene encoding the SCoAL β subunit, which can complement mutants with α-subunit and β-subunit defects in *Saccharomyces cerevisiae*, respectively [23]. Although the phenotype, photosynthetic rate, and respiration rate of plants do not change significantly after antisense suppression or RNA interference of the SCoAL β subunit-encoding gene, the production of succinic acid from γ-aminobutyric acid is upregulated [24]. Transgenic tomato plants expressing *SlSDH2-2* antisense show higher plant height, fruit weight, and photosynthetic rate, but the flux of the TCA cycle is reduced [25]. Antisense inhibition of *SlFUM1* results in a decrease in TCA cycle flux and photosynthesis rate, a slight delay in root growth of transgenic plants, and a decrease in fruit yield [26]. Antisense inhibition of *SlmMDH* results in enhanced photosynthesis and reduced respiration rate, and plants exhibit a faster growth rate and higher dry matter accumulation [27]. Antisense inhibition of either *SlmMDH* or *SlFUM1* in tomatoes causes a dramatic decrease in root dry matter deposition and respiratory activity but opposite changes in the root area, transitory starch content and soluble sugar content [28, 29]. A mild reduction in CSY activity results in impaired nitrate assimilation and reduced leaf pigmentation but has no significant effect on photosynthetic performance or plant growth [30].

The biological functions of a few TCA cycle genes of potato have also been identified. Antisense inhibition of cytoplasmic NADP-specific isocitrate dehydrogenase does not cause significant changes in the growth, development, photosynthetic efficiency, or respiration rate of potato plants [31]. *StFum-1* has a mitochondrial targeting peptide and can complement a fumarase-deficient *Escherichia coli* mutation [32]. Antisense inhibition of the *StCSY* gene results in the abortion of flower buds, which cannot develop into mature flowers [33]. Inhibition of 2-oxoglutarate dehydrogenase in potato tuber via the use of specific in vivo inhibitors suggests that αKGDHC limits respiration and plays an important role in nitrogen assimilation [34].

The above findings indicate that TCA cycle genes play important functions in the growth and development of Solanaceae crops. To further reveal the composition and functional characteristics of TCA cycle genes in Solanaceae crops, this study identified the TCA cycle genes in the genomes of four important Solanaceae vegetable crops, potato, tomato, eggplant, and pepper, and compared and analyzed their phylogenetic characteristics, chromosome collinearity, and tissue expression patterns. The expression characteristics of the potato TCA cycle genes and the subcellular localization of the three CSYs in potato were analyzed.

## Results

### TCA gene identification

**Fig. 1:**
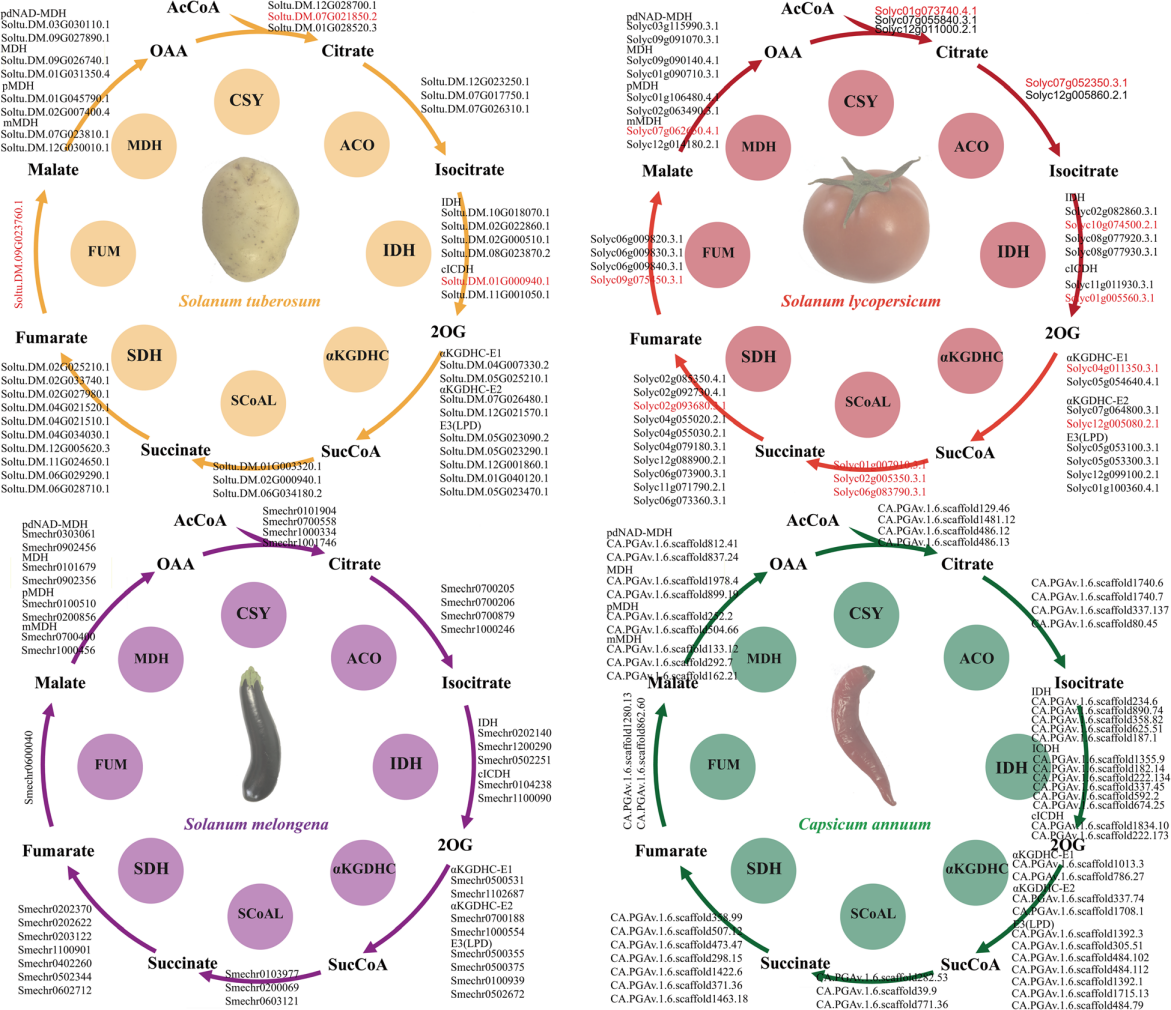
Distribution of TCA cycle genes in potato, tomato, eggplant, and pepper. Circles represent enzyme complexes. Based on the highest score in the BLASTP analysis, the query genes were anchored to each TCA cycle complex or subunit. Characterized TCA cycle genes in Solanaceae are shown in red font. ACO, cis-aconitase; IDH, isocitrate dehydrogenase; αKGDH, α-ketoglutarate dehydrogenase; SCoAL, succinyl-CoA ligase; SDH, succinate dehydrogenase; FUM, fumarate hydratase; MDH, malate dehydrogenase; CSY, citrate synthase

**Fig. 2:**
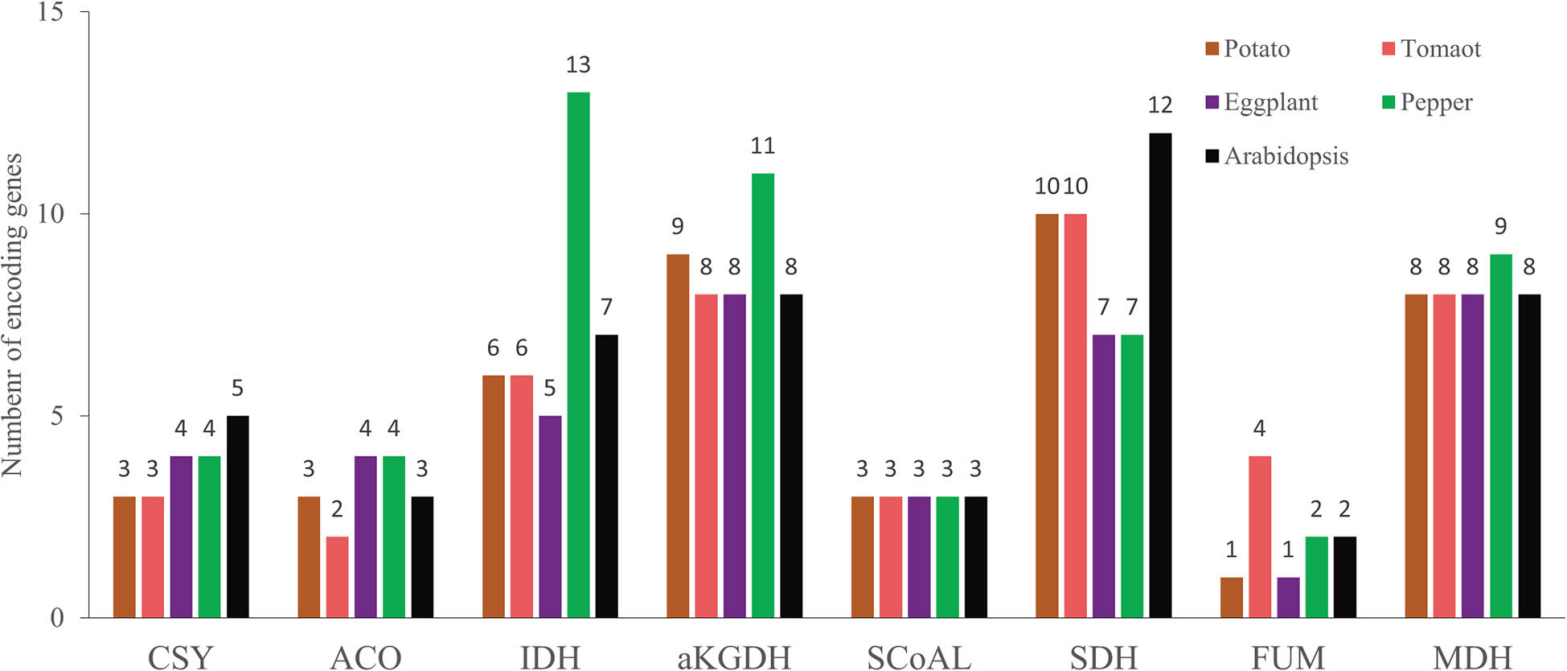
Comparison of the number of TCA cycle genes between the five species. The abscissa represents various TCA enzyme complexes, and the ordinate represents the number of genes

Based on the BLAST comparison of Arabidopsis TCA cycle proteins, 43, 44, 40, and 53 TCA cycle genes were identified in potato, tomato, eggplant, and pepper, respectively (Fig. 1 and Additional file 1). The number of genes of a given enzyme complex was approximately the same between species. In particular, pepper had the most candidate genes for αKGDHC, IDH, and MDH, and tomato had the most candidate genes for FUM. Moreover, compared with Arabidopsis, Solanaceae crops had the same number of SCoAL-encoding genes but fewer members of the CSY and SDH enzyme complexes (Fig. 2). In summary, the number of genes that constituted the TCA cycle was approximately the same between Solanaceae crops and was particularly more conserved among potato, tomato, and eggplant.

### Phyletic evolution and collinearity analysis

**Fig. 3:**
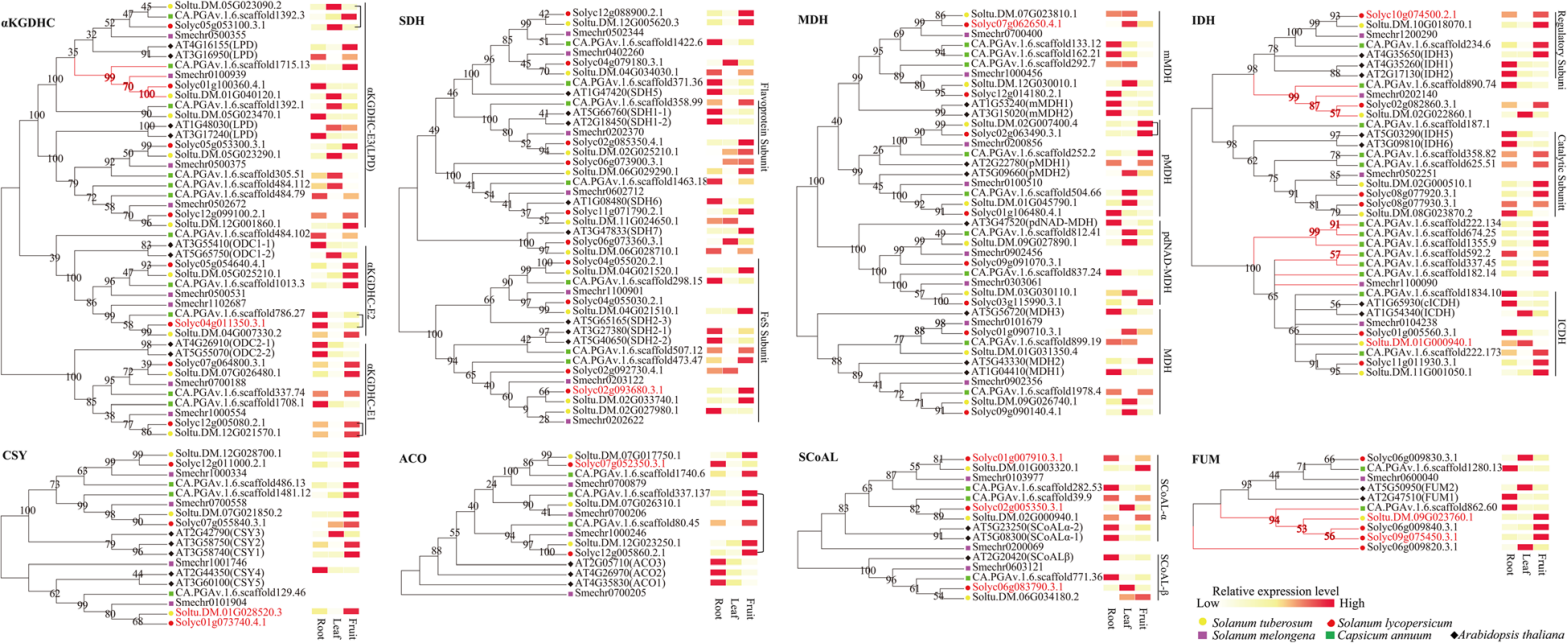
Evolution and expression analysis of TCA cycle genes in potato, tomato, eggplant, pepper, and Arabidopsis. Red branches indicate homologous genes unique to Solanaceae crops. Characterized TCA cycle genes in Solanaceae are shown in red font. There was a positive correlation (*p* ≤ 0.05) between Solanaceae crop expression levels, and orthologous gene pairs were connected by straight lines. Gene expression data were obtained from Spud DB (http://potato.plantbiology.msu.edu/, potato), TomExpress (http://tomexpress.toulouse.inra.fr/, tomato), and TRAVA (http://travadb.org/, Arabidopsis). The TCA gene expression data of pepper were obtained from Kim et al. [65]. The expression characteristics of each gene were evaluated by Z-score

Based on the TCA cycle protein sequences of the four Solanaceae crops and Arabidopsis, a phylogenetic tree of each enzyme complex was constructed (Fig. 3). The results showed that, in addition to division by ACO, CSY, and FUM, the presence of enzyme subunits led to the subdivision of proteins of other enzyme complexes into multiple subgroups, and each subgroup contained members of all five species. Importantly, compared to Arabidopsis, Solanaceae had four unique evolutionary branches, including one each for αKGDHC and FUM and two for IDH. In addition, except for one branch in FUM and IDH, the specific branches of Solanaceae crops were often composed of genes of all four Solanaceae crops, indicating that the founding members of these branches may have arisen before the differentiation of Solanaceae species.

**Fig. 4:**
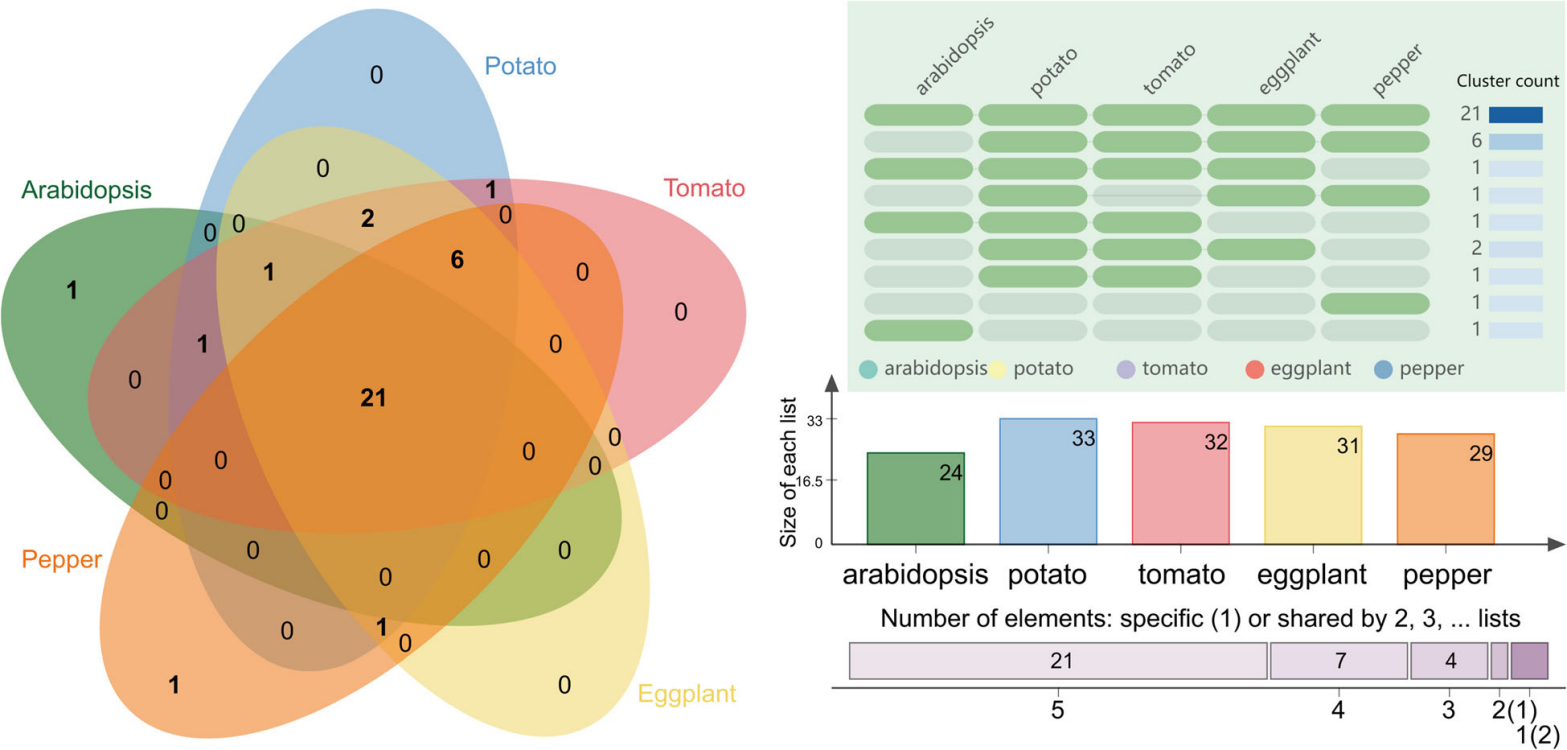
Orthologous clusters of TCA cycle genes in potato, tomato, eggplant, pepper, and Arabidopsis

Homologous gene clusters of the TCA cycle proteins of potato, tomato, eggplant, pepper, and Arabidopsis were identified (Fig. 4). These TCA cycle proteins formed 35 gene clusters (Additional file 2), of which 21 orthologous gene clusters contained genes from all five species. Seven homologous gene clusters consisted of genes from four species, six of which were Solanaceae-specific. On the other hand, the identification results of paralogous genes (Additional file 3) showed that there were more paralogous genes in Arabidopsis (15 pairs) than in pepper (11 pairs), and the number of paralogous genes in eggplant (four pairs), potato (four pairs), or tomato (three pairs) was relatively small. In summary, the identification of orthologous and paralogous gene sets showed that the TCA cycle genes are highly conserved among Solanaceae crop species.

**Fig. 5:**
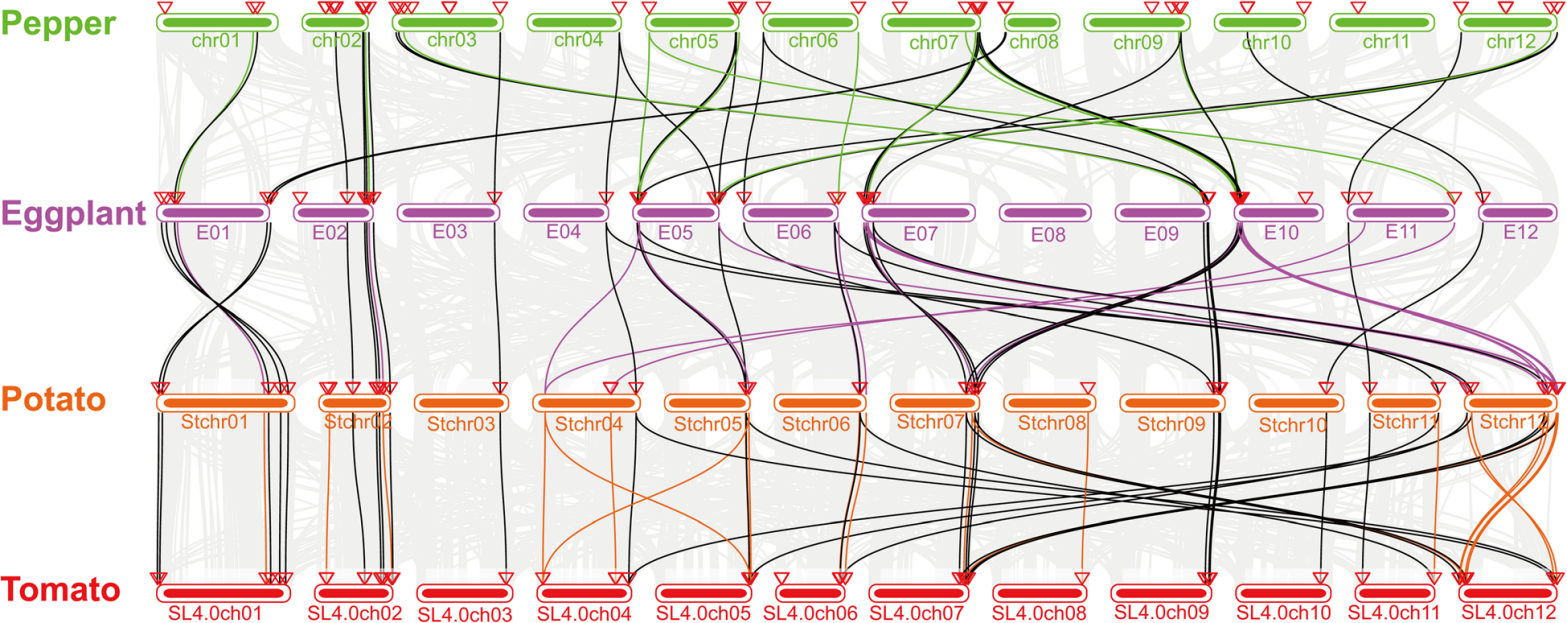
Collinearity analysis of TCA cycle genes in Solanaceae crops. Gray: collinearity of the TCA cycle genes between two neighboring Solanaceae crop genomes; green: pepper and eggplant; purple: eggplant and potato; orange: potato and tomato; black: all four Solanaceae crops. The triangles indicate the position of each TCA cycle gene on the chromosome

Based on the collinearity analysis of TCA cycle genes between paired Solanaceae crops (Figs. 5), 32 (60.4 %) TCA cycle genes in pepper had collinearity with tomato, 34 (85.0 %) TCA cycle genes in eggplant had collinearity with the potato genome, and 36 (83.7 %) TCA cycle genes in the potato had collinearity with tomato genomes. Twenty-one TCA cycle genes showed collinearity between all four Solanaceae. In summary, collinearity analysis indicated that the chromosomal location of the TCA cycle genes has been relatively conserved in the evolution of Solanaceae crops. In addition, we noticed that TCA cycle genes in Solanaceae were not randomly distributed on chromosomes but tended to be located at either end of a chromosome, and some genes appeared in clusters on chromosomes.

### Comparison of TCA cycle gene expression patterns between species

Based on public expression data, we conducted a comparative analysis of the TCA cycle gene expression patterns among potato, tomato, pepper, and Arabidopsis (Fig. 3). We found that (1) CSY genes in the four species were mainly expressed in the fruits; (2) IDH genes of the four species were mainly expressed in roots or fruits, while the expression levels in leaves were low; (3) ACO enzyme complex genes in Solanaceae crops were mainly expressed in fruits, while Arabidopsis ACO genes were mainly expressed in roots; and (4) other enzyme complex–encoding genes were expressed in roots, leaves, and fruits. Orthologous genes tended to have similar tissue expression patterns, and the expression patterns of the five orthologous gene pairs encoding αKGDH, ACO, and MDH showed positive correlations between Solanaceae crops (Fig. 3). These findings suggest that the functions of these genes are more conserved in Solanaceae crops.

### Analysis of the tissue expression of potato TCA genes

**Fig. 6:**
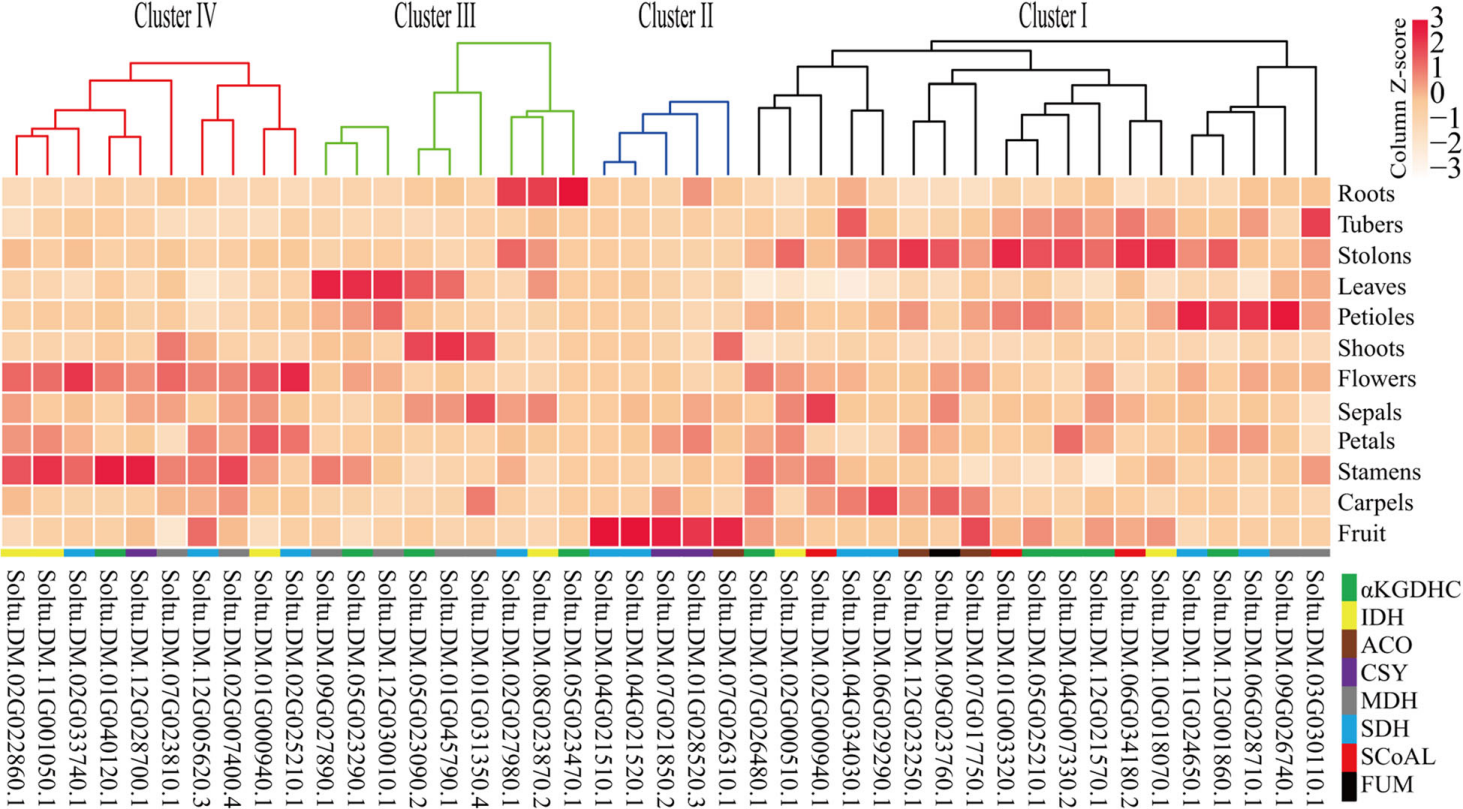
Expression of the TCA cycle genes in different organs of potato. The expression data (reads per kilobase per million reads value) were downloaded from the Spud DB database and normalized by Z-score

**Fig. 7:**
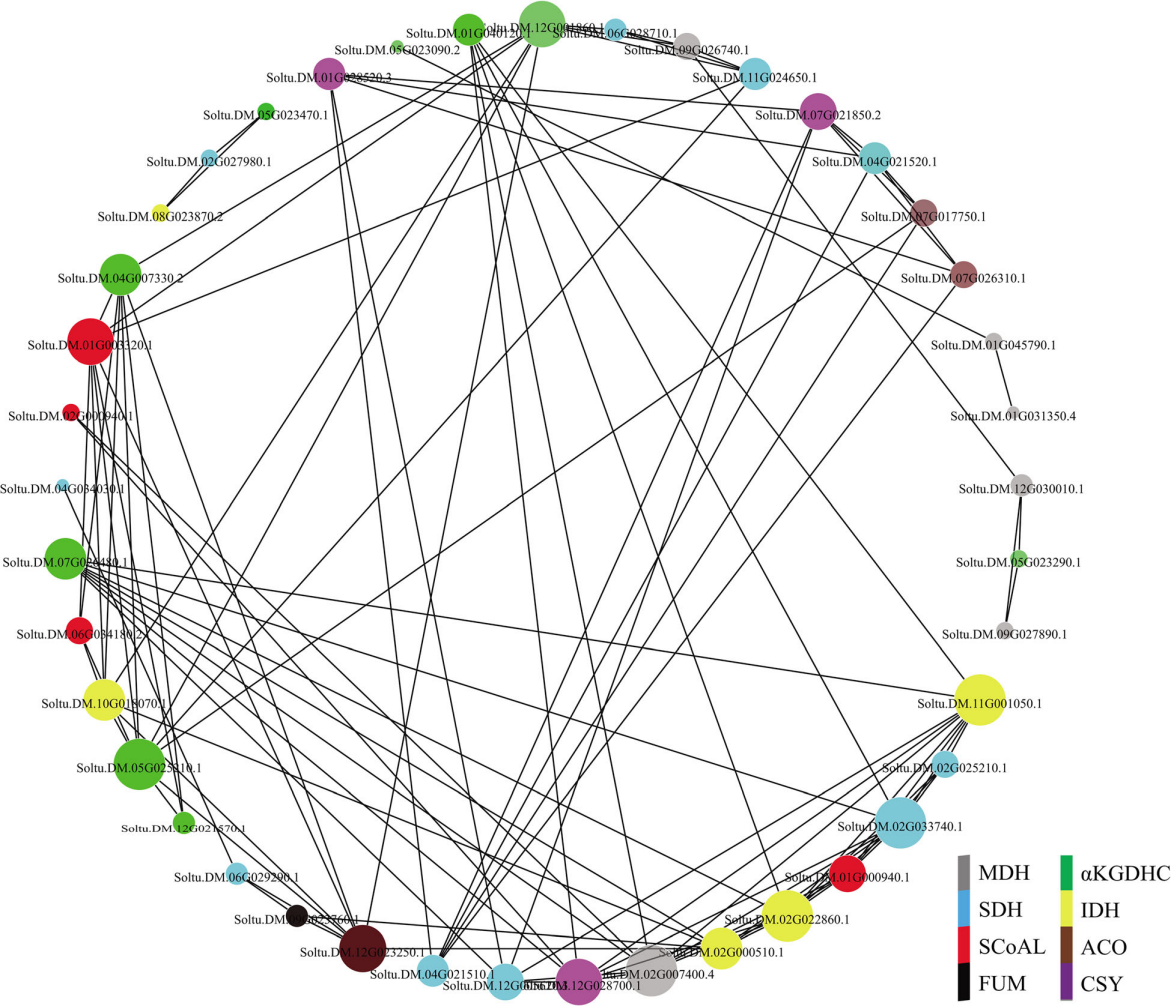
Coexpression analysis of TCA cycle genes in potato. Only the coexpressed genes with a positive correlation (*p* ≤ 0.05) are shown in the figure. The circles with different colors indicate different enzymes, and the size of the circle corresponds to the number of coexpressed genes

TCA cycle genes were expressed in various potato tissues (Fig. 6). Some genes from the same enzyme complex had similar tissue expression patterns, including Soltu.DM.12G021570.1, Soltu.DM.04G007330.2, and Soltu.DM.05G025210.1 in the αKGDH enzyme complex; Soltu.DM.01G031350.4, Soltu.DM.01G045790.1, Soltu.DM.03G030110.1, and Soltu.DM.09G026740.1 in the MDH enzyme complex; Soltu.DM.04G021520.1, Soltu.DM.04G021510.1, Soltu.DM.06G029290.1, and Soltu.DM.04G034030.1 in the SDH enzyme complex; Soltu.DM.11G001050.1 and Soltu.DM.02G022860.1 in the IDH enzyme complex; and Soltu.DM.01G028520.3 and Soltu.DM.07G021850.2 in the CSY enzyme complex. According to the characteristics of gene expression in tissues and organs, potato TCA cycle genes could be roughly divided into four groups: Cluster I contained the most genes, and their expression levels in tubers and stolons were higher; the five cluster II genes were relatively highly expressed in the fruit; the cluster III genes were relatively highly expressed in vegetative organs such as potato roots, leaves, and stems; and the cluster IV genes were mainly expressed in the reproductive organs. The correlation between the expression patterns of TCA cycle genes in potato was analyzed (Fig. 7). The results showed that a total of 100 pairs of TCA cycle genes had a significant positive correlation (*p* ≤ 0.05), of which 20 pairs of coexpressed genes were from the same enzyme complex, while the other 80 pairs of coexpressed genes were from different enzyme complexes. Five genes, Soltu.DM.11G001050.1 (IDH), Soltu.DM.02G022860.1 (IDH), Soltu.DM.02G033740.1 (SDH), Soltu.DM.02G007400.4 (MDH), and Soltu.DM.05G025210.1 (αKGDH), had the most coexpression partners (nine each), suggesting their functional diversity.

### Hormone-induced expression of TCA cycle genes in potato

**Fig. 8:**
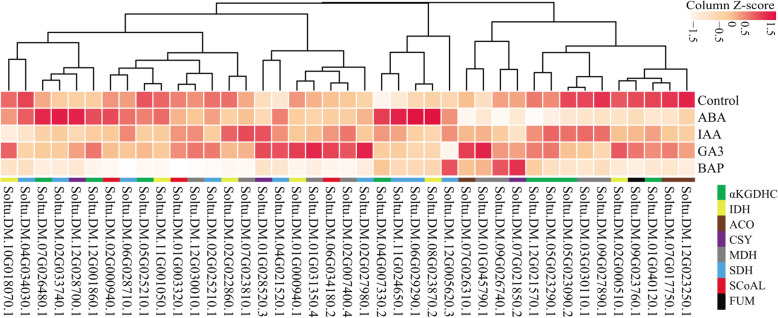
Response analysis of the potato TCA genes to IAA, GA3, BAP, and ABA

As shown in Fig. 8, compared to the control group, most TCA cycle genes (excluding Soltu.DM.07G021850.2, Soltu.DM.09G026740.1, and Soltu.DM.12G005620.3) underwent a decrease in expression level after treatment with the cytokinin-like substance BAP. Under auxin treatment, the expression levels of TCA cycle members in potato were downregulated or did not change significantly, while Soltu.DM.01G045790.1, Soltu.DM.07G026310.1, Soltu.DM.02G027980.1, Soltu.DM.01G031350.4, and Soltu.DM.01G000940.1 were specifically induced by GA3. Under ABA treatment, the expression levels of eight genes, Soltu.DM.04G007330.2, Soltu.DM.11G024650.1, Soltu.DM.06G029290.1, Soltu.DM.08G023870.2, Soltu.DM.07G026480.1, Soltu.DM.02G033740.1, Soltu.DM.12G028700.1, and Soltu.DM.12G001860.1, were increased. The above results indicate that members of the potato TCA cycle are involved in the response to hormones such as ABA, gibberellin, and cytokinin.

### Analysis of the disease response of potato TCA genes

**Fig. 9:**
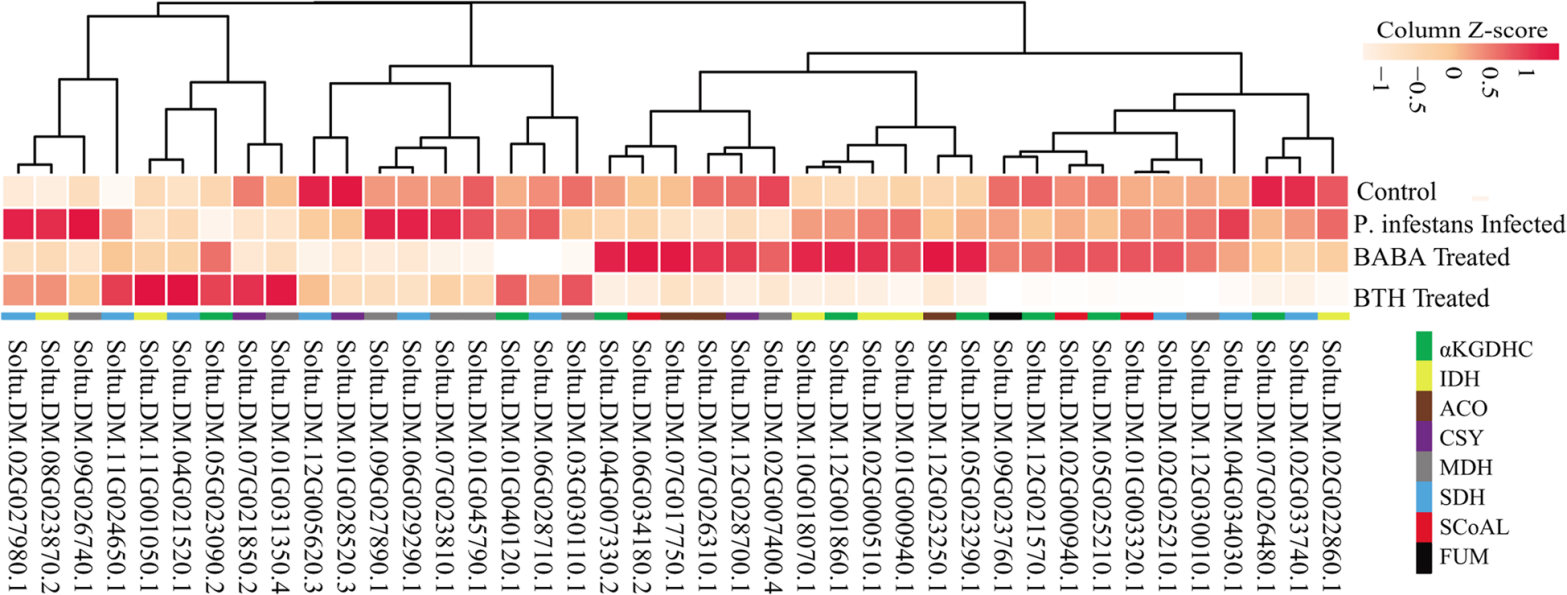
Expression analysis of TCA genes in potato treated with *Phytophthora infestans*, BABA, and BTH. BABA, dl-β-amino-*n*-butyric acid; BTH, acibenzolar-S-methyl

*Phytophthora infestans* causes the main disease that potatoes suffer from in production. In this study, we found that expression levels of some TCA cycle genes, including Soltu.DM.02G027980.1, Soltu.DM.08G023870.2, and Soltu.DM.09G026740.1, were significantly increased after plants were infected with *P. infestans* (Fig. 9). DL-β-Amino-*n*-butyric acid (BABA) is a secondary metabolic nonprotein amino acid that can induce disease resistance. BABA mainly induced the upregulation of Soltu.DM.04G007330.2, Soltu.DM.06G034180.2, Soltu.DM.07G017750.1, Soltu.DM.10G018070.1, Soltu.DM.12G001860.1, Soltu.DM.02G000510.1, Soltu.DM.01G000940.1, Soltu.DM.12G023250.1, and Soltu.DM.05G023290.1. Acibenzolar-S-methyl (BTH) is a typical plant disease resistance inducer that induced the upregulation of Soltu.DM.01G031350.4, Soltu.DM.11G001050.1, Soltu.DM.04G021520.1, and Soltu.DM.11G024650.1. Importantly, the level of Soltu.DM.11G024650.1 can be simultaneously upregulated by the three disease resistance inducers. The above results indicate that the TCA cycle genes may be involved in the disease resistance of potato.

### Analysis of the involvement of potato TCA cycle genes in osmotic regulation

**Fig. 10:**
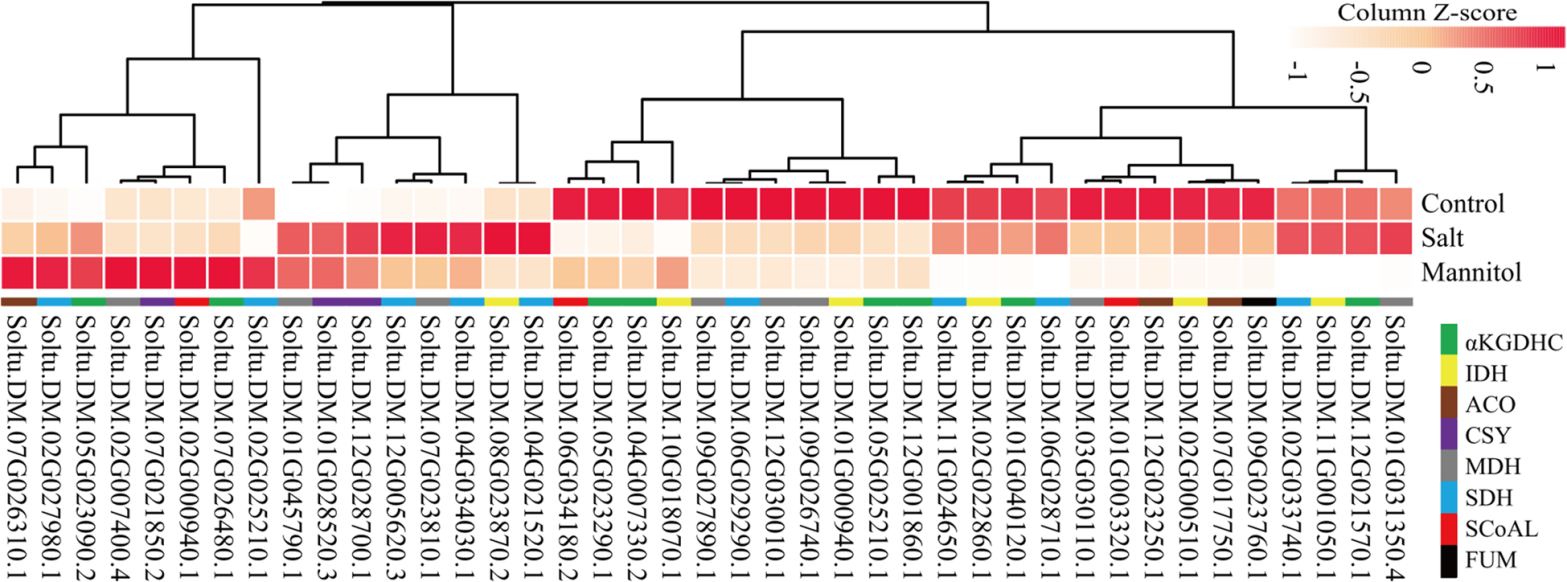
Expression levels of the TCA cycle genes in potato treated with salt and mannitol

As shown in Fig. 10, under salt and mannitol treatments, we found that the expression of TCA cycle genes was changed. These genes can be divided into two types: In one type, gene expression levels were elevated under osmotic stress, in which the expression levels of Soltu.DM.01G045790.1, Soltu.DM.01G028520.3, and Soltu.DM.12G028700.1 were increased under salt and mannitol treatments. The other category included most genes, whose expression levels were suppressed under osmotic stress.

### Analysis of potato CSY sequences and subcellular localizations

**Fig. 11:**
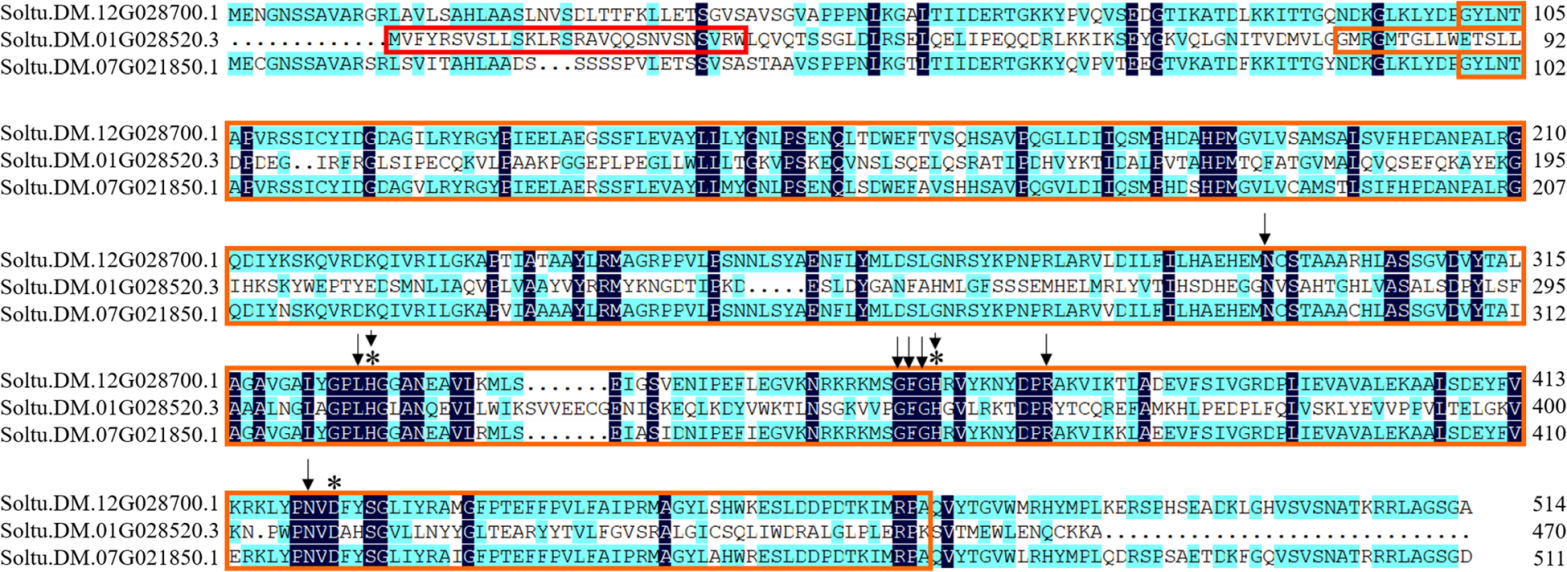
Alignment analysis of the amino acid sequences of three potato CSYs. The orange color represents the functional domain of CSY (pfam00285), and the red color represents the predicted putative mitochondrial localization presequences. The conserved functional sites include the coenzyme binding sites, marked with arrows, and the amino acids of the catalytic triad, marked with asterisks

**Fig. 12:**
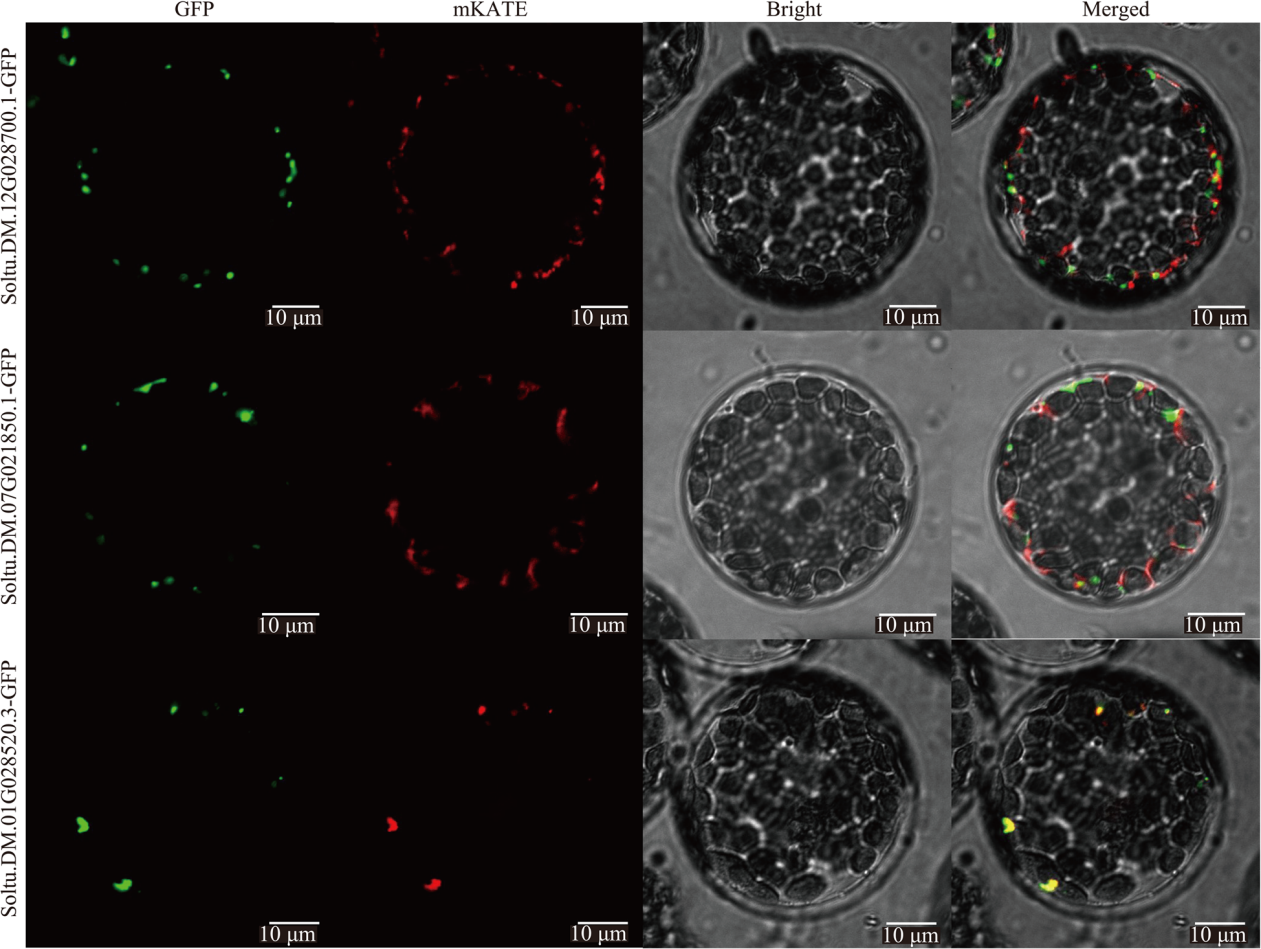
Colocalization analysis of three potato CSY proteins with mitochondria. The green fluorescence of GFP is the fusion protein fluorescence, the red fluorescence of mKATE is the mitochondrial marker, and the yellow fluorescence is the overlap of the two

CSY is the first rate-limiting enzyme of the TCA cycle and the main regulatory target in the TCA cycle. We compared the sequence characteristics and subcellular localizations of the three CSY candidate genes in potato. The sequence alignment results showed that the amino acid sequence identity between Soltu.DM.12G028700.1 and Soltu.DM.07G021850.1 was more than 85 %, while the amino acid sequence identity between these two and Soltu.DM.01G028520.3 was only approximately 15 %. The prediction of conserved domains showed that all three genes had conserved residues, such as typical coenzyme binding sites and the catalytic triad site (His–His–Asp) (Fig. 11). The subcellular prediction results showed that Soltu.DM.01G028520.3 had mitochondrial targeting sequences and could be localized in mitochondria, while Soltu.DM.12G028700.1 and Soltu.DM.07G021850.1 did not have mitochondrial targeting signals and might be localized to peroxisomes. Furthermore, we separately fused the three CSY candidate genes from potato to GFP and transiently expressed them in Arabidopsis protoplasts. The results showed that the Soltu.DM.01G028520.3–GFP fusion protein completely overlapped with the mitochondria, indicating that this CSY mainly functions in mitochondria (Fig. 12). In contrast, the green fluorescence of the Soltu.DM.12G028700.1 and Soltu.DM.07G021850.1 fusion proteins did not overlap with the corresponding red fluorescence of the mitochondrial marker, so their localization in the cytoplasm needs to be further investigated. Based on this analysis of subcellular localization, we hypothesize that Soltu.DM.01G028520.3 is more likely to be directly involved in the potato TCA cycle in mitochondria.

## Discussion

The TCA cycle has important application potential in improving crop yield [2]. TCA cycle genes are extensively involved in the plant growth and development processes, such as seed germination [35], number and weight [36, 37], root development [38], leaf development and senescence [21, 39], flower development [33, 39, 40], and fruit ripening [21, 29]. By whole-genome identification, this study identified 43, 44, 40, and 53 TCA cycle members in four important Solanaceae vegetable crops, potato, tomato, eggplant, and pepper, respectively. The phylogenetic, collinearity, and tissue expression pattern relationships between the TCA cycle genes in four Solanaceae crops were analyzed, and the evolutionary characteristics and possible biological functions of the TCA genes were revealed. We further analyzed the expression patterns of TCA cycle members in different potato tissues and their responses to biotic and abiotic stresses. In addition, the sequences and subcellular localizations of three potato CSYs were compared. The results of this study provide reference information for further understanding the biological functions of TCA cycle genes in Solanaceae crops.

Compared to the 48 TCA cycle genes in Arabidopsis, there were varying numbers of TCA cycle members in the genomes of the four Solanaceae crops. Based on our phylogenetic analysis (Fig. 3), αKGDH, IDH, FUM, and other catalytic enzymes formed four branches unique to Solanaceae crops, of which two branches contained genes from all four Solanaceae crops, suggesting these TCA cycle genes have functional differentiation between Arabidopsis and Solanaceae crops. Compared with other species, pepper has more IDH-encoding genes, and it is necessary to further analyze their functions in the TCA cycle and in the growth and development of pepper. In contrast to Arabidopsis and potato, which have only one FUM-encoding gene, there were four FUM isoenzymes in tomato, which is consistent with a previous study that used the entire coding region of *SlFUM1* to perform Southern hybridization against the tomato genome and detected multiple homologous fragments [26]. In plants, the succinate dehydrogenase complex (complex II) is composed of eight subunits, of which the amino acid sequences of SDH1 and SDH2 are conserved among different species, while the other subunit sequences are not conserved [41]. This is why we did not identify the homologs of Arabidopsis SDH3, SDH4, SDH7, and SDH8 in Solanaceae crops in our homology analysis and explains why there were more SDH enzyme complex genes in Arabidopsis than in Solanaceae crops.

Previous studies have shown that genes involved in plant secondary metabolism tend to cluster on chromosomes [42–44]. The tricarboxylic acid cycle is the primary metabolic process in cells. We found that some of the TCA cycle genes in Solanaceae appear to be clustered in certain chromosomes, which is very interesting. However, after careful examination of each location of these TCA genes on the chromosome, it was found that the distance between them was relatively great, and they were often separated by other genes, which did not meet the existing criteria for identification as a gene cluster [45, 46].

The TCA cycle genes are sensitive to environmental stressors such as osmotic conditions, salt, oxidative state, UV-B radiation, flooding, and cold [5, 47–51]. At the same time, several plant TCA cycle members assist plants in fighting against pathogens [20, 22, 29, 52]. The roles of the TCA cycle genes in potato growth and development remain largely unclear. Based on the expression analysis, we found that the potato TCA cycle genes may play a role in the hormone response, disease resistance, and osmotic regulation. The transcriptome data showed that the TCA cycle members of potato actively responded to ABA and GA and showed a trend of downregulation after cytokinin treatment. Plant endogenous hormones can regulate the expression of mitochondrial genes either directly through anterograde signaling or indirectly by affecting retrograde signaling and can even directly bind to mitochondrial proteins to affect their functions [53]. For example, ABA can directly affect the metabolic activity of the TCA cycle, and TCA cycle flux is significantly increased in the Arabidopsis ABA signaling factor mutant *srk2d srk2e srk2i* [54]. The involvement of the TCA cycle in the disease resistance of Solanaceae crops has been reported. For example, the αKGDHC E2 subunit of tomato can directly bind to the plant defense signaling molecule salicylic acid and play a negative regulatory role in the resistance to TMV infection [11]. The concentrations of intermediate metabolites of the TCA cycle, such as α-ketoglutarate, citric acid, and succinic acid, are significantly changed after plant inoculation with potato virus Y [55]. Mitochondria, especially via the TCA cycle, play a crucial role in the abiotic stress responses of plants [56]. In this study, most TCA cycle genes in potato were downregulated under mannitol and NaCl treatments, which may be a manifestation of the active adaptation of cellular energy metabolism to osmotic stress [57] or of the physiological and chemical inhibition by salt [58].

CSYs catalyze the production of citryl-CoA from acetyl-CoA and oxaloacetate, which is hydrolyzed to produce citric acid and coenzyme A [59]. One CSY was obtained from the potato cultivar Desiree based on homologous cloning, which can restore the CSY activity of *E. coli* mutant strain K214 [60]. Antisense interference with this CSY results in failed potato flower formation [33]. Sequence alignment showed that the amino acid sequence of Soltu.DM.01G028520.3 analyzed in this study was 91.75 % identical to the previously reported CSY. Moreover, the results of our subcellular localization assay showed that Soltu.DM.01G028520.3 could indeed target the mitochondria. Therefore, Soltu.DM.01G028520.3 appears to be the major mitochondrial CSY isoform in potato.

## Conclusions

Based on homologous alignment, we identified members of the TCA cycle in four Solanaceae vegetables: potato, tomato, eggplant, and pepper. Tomato had more FUM enzyme genes, while pepper had more IDH-encoding genes. The evolutionary relationship, collinearity, and tissue expression pattern of the TCA cycle genes in these four Solanaceae vegetables were determined. Compared to Arabidopsis, Solanaceae had unique members of the TCA subgroup. Importantly, tissue expression analysis combined with biotic and abiotic stress response expression analysis identified the key TCA cycle genes in the potato’s response to hormones, disease, and osmotic stress. In addition, we compared the sequence characteristics and subcellular localizations of three potato CSY isozymes, revealing the key role of Soltu.DM.01G028520.3 in the production of citric acid.

## Methods

### TCA cycle gene identification

The 48 genes directly involved in the TCA cycle in Arabidopsis were used as decoys [6, 61]. Candidate TCA cycle genes were identified genome-wide in potato, tomato, eggplant, and pepper using localized BLASTP with a threshold value of e-value < 1e-30. Genomic data and annotation information for potato (DM v6.1), tomato (ITAG4.0), eggplant (SME-HQ), and pepper (Annuum.v. 2.0) were downloaded from the Spud DB (http://potato.plantbiology.msu.edu/) [62], SGN (https://ftp.solgenomics.net/) [63], Eggplant Genome Database (http://eggplant-hq.cn/Eggplant/home/index) [64], and PEPPER GENOME databases (http://peppergenome.snu.ac.kr/) [65]. For the same target gene, only the best hit was retained for subsequent analysis. The conserved structural domains of all obtained candidate genes were identified (e-value < 0.01) by the online tool NCBI CDD V3.19 (https://www.ncbi.nlm.nih.gov/cdd) [66], and genes that did not contain conserved functional domains were excluded.

### Analysis of system evolution

The TCA cycle protein sequences of potato, tomato, eggplant, pepper, and Arabidopsis were subjected to multiple sequence alignment using ClustalX [67], then submitted to MEGA 5.10 [68] to construct a phylogenetic tree using the neighbor-joining method, with a bootstrap value of 1,000. All TCA cycle protein sequences were submitted to the online software OrthoVenn2 (https://orthovenn2.bioinfotoolkits.net/home) [69] to identify the orthologs and paralogs. All the parameters were set to default values. In addition, based on the downloaded genomic information, the collinearity of TCA members between Solanaceae crops was plotted (e-value <1e-10) by TBtools [70] using MCScanX [71].

### Analysis of cross-species tissue expression patterns

To preliminarily investigate the tissue expression characteristics of TCA cycle genes in potato, tomato, pepper, and Arabidopsis, TCA cycle gene expression data from roots, leaves, and fruits of potato, tomato, and Arabidopsis were downloaded from Spud DB, TomExpress (http://tomexpress.toulouse.inra.fr/) [72], and TRAVA (http://travadb.org/) [73], respectively. The TCA cycle gene expression data of pepper were from publicly available transcriptome data [65]. The above TCA cycle gene expression data were submitted to an online platform named Omicshare (http://www.omicshare.com/tools), and the correlation analysis of the expression levels of orthologous genes among Solanaceae species was analyzed through the Pearson correlation coefficient (PCC) with default values of parameters in Omicshare. The orthologous and paralogous gene pairs were identified using the online software OrthoVenn2 (https://orthovenn2.bioinfotoolkits.net/home) with default values.

### Tissue expression profile analysis of potato TCA cycle genes

The expression data (fragments per kilobase million value, FPKM) of the TCA cycle genes in the vegetative and reproductive tissues of potato double haploid DM1-3 516R44 were obtained from the Spud DB database (http://potato.plantbiology.msu.edu/). These tissues were roots, tubers, stolons, leaves, petioles, shoots, flowers, sepals, petals, stamens, carpels, and fruits [74, 75]. After normalization by the Z-score, the expression patterns of each gene in various potato tissues were compared and analyzed. We submitted the above TCA cycle gene expression data to Omicshare. The correlation analysis of the expression levels of the genes was calculated as the Pearson correlation coefficient with default Omicshare parameter values.

### Analysis of the response of potato TCA cycle genes to abiotic stress

The expression profiles of TCA cycle genes in potato plants treated with various hormones or abiotic stressors were downloaded from Spud DB [74, 75]. The gene expression data were obtained from transcriptome sequencing of root and shoot mixtures after treating of the potato double haploid DM1-3 516R44 with stressors under the environmental conditions of a 16-hour photoperiod and temperature of 22 °C day/18°C night for 24 h. The hormone treatments included 10 µM indoleacetic acid (IAA), 50 µM gibberellic acid (GA3), 10 µM 6-benzylaminopurine (BAP), and 50 µM abscisic acid (ABA). The abiotic stress conditions included 150 mM NaCl and 260 µM mannitol.

### Analysis of the response of potato TCA cycle genes to biological stress

To identify whether TCA cycle genes are involved in disease resistance in potato, we analyzed changes in the expression levels of the TCA gene in leaves after infection with *P. infestans* or treatment with the chemical inducers BTH and BABA. Six leaves were collected from the DM1-3 516R44 potato plant grown in the greenhouse and inoculated with 0.5-0.7 mL of *P. infestans* (Pi isolate US8: Pi02-007) at a concentration of 30,000 sporangia/mL by spraying. Meanwhile, 100 mg/mL BTH and 2 mg/mL BABA were sprayed to simulate the inoculation of pathogens. The inoculated leaves were stored at room temperature in the dark for 8–10 h and then cultured in light for 7 days. The infection experiment was repeated three times. Leaves were collected 24, 36, and 72 h after inoculation for transcriptome sequencing of pooled DNA samples (pool-seq). Transcriptome data were downloaded from Spud DB [74, 75].

### Colocalization analysis of potato CSYs and mitochondria

Amino acid sequences of three CSYs (Soltu.DM.01G028520.3, Soltu.DM.12G028700.1, and Soltu.DM.07G021850.1) were obtained by referring to the annotation information in Spud DB. The online software Plant-mPLoc [76] was used to predict the subcellular localizations of the three CSYs, and then, MitoFates [77] was used to predict the mitochondrial targeting sequences. Due to the update of the Spud DB database, the expression profile analysis mainly focused on another splicing form, Soltu.DM.07G021850.2. Furthermore, we constructed coding sequence–enhanced green fluorescent protein (GFP) fusion expression vectors of three CSY genes driven by the 35S rRNA promoter separately and further verified the subcellular localization of the three CSY genes by confocal microscopy. Briefly, protoplasts of Arabidopsis seedlings were transformed with Agrobacterium containing the fusion expression vector by polyethylene glycol induction and cultured in a dark room at 28 °C for 24-48 h, and then fluorescence was observed under a Nikon C2-ER laser confocal microscope. In this experiment, the excitation wavelength of GFP was 488 nm, and the emission wavelength was 510 nm. 35S::mtsp-mKATE was used as a mitochondrial marker. The excitation wavelength of the red fluorescent protein mKATE was 561 nm, and the emission wavelength was 580 nm. Finally, the open-source software ImageJ (https://imagej.nih.gov/ij/) [78] was used to perform colocalization analysis of the target proteins and mitochondria.

## Supplementary information

Additional file 1 The TCA cycle proteins in four Solanaceous crops.

Additional file 2 Detailed list of orthologous gene clusters and singletons between *Solanum tuberosum*, *Solanum lycopersicum*, *Solanum melongena*, *Capsicum annuum*, and *Arabidopsis thaliana*.

Additional file 3 Detailed list of paralogous gene pairs in *Solanum tuberosum*, *Solanum lycopersicum*, *Solanum melongena*, *Capsicum annuum*, and *Arabidopsis thaliana*.

## Data Availability

All data generated or analyzed during this study are included in this article and its additional files. The genome data of potato was downloaded from http://potato.plantbiology.msu.edu/. The genome data of tomato were downloaded from https://ftp.solgenomics.net/. The genome data of eggplant were downloaded from http://eggplant-hq.cn/Eggplant/home/index. The genome data of pepper were downloaded from http://peppergenome.snu.ac.kr/. The TCA cycle gene expression data of potato, tomato, and Arabidopsis were downloaded from Spud DB (http://potato.plantbiology.msu.edu/), TomExpress (http://tomexpress.toulouse.inra.fr/ ), and TRAVA (http://travadb.org/), respectively. The TCA cycle gene expression data of pepper were from publicly available transcriptome data [65].

## Abbreviations

ABA: abscisic acid
ACO: aconitase
αKGDHC: α-ketoglutarate dehydrogenase complex
BABA: DL-β-amino-n-butyric acid (BABA)
BAP: benzylaminopurine
BTH: acibenzolar-S-methyl
CSY: citrate synthase
GA: gibberellic acid
GFP: green fluorescent protein
FPKM: fragments per kilobase million value
FUM: fumarase
IAA: indoleacetic acid
IDH: isocitrate dehydrogenase
MDH: malate dehydrogenase
NAD(P): nicotinamide adenine dinucleotide/nicotinamide adenine dinucleotide phosphate
PCC: Pearson correlation coefficient
SCoAL: succinyl-CoA synthetase
SDH: succinate dehydrogenase
TCA cycle: tricarboxylic acid cycle
TMV: tobacco mosaic virus
VIGS: virus-induced gene silencing
